# Supplementary material for: Efficient Antibacterial/Antifungal Activities: Synthesis, Molecular Docking, Molecular Dynamics, Pharmacokinetic, and Binding Free Energy of Galactopyranoside Derivatives
Source: Molecules. 2022 Dec 26;28(1):219. doi: 10.3390/molecules28010219 (PMC9822152; doi:10.3390/molecules28010219)
Supplement: Supplementary file 1 [file molecules-28-00219-s001.zip › molecules-1978757-supplementary.pdf]

molecules

MDPI

Supplementary Materials

Efficient Antibacterial/Antifungal Activities: Synthesis, Molecular Docking, Molecular Dynamics, Pharmacokinetic, and Binding Free Energy of Galactopyranoside Derivatives

Faez Ahmmed [1], Anis Ul Islam [1], Yousef E. Mukhrish [2], Youness El Bakri [3], Sajjad Ahmad [4], Yasuhiro Ozeki [5] and Sarkar M. A. Kawsar [1,*]

[1] Laboratory of Carbohydrate and Nucleoside Chemistry (LCNC), Department of Chemistry, Faculty of Science, University of Chittagong, Chittagong-4331, Bangladesh

[2] Department of Chemistry, Faculty of Science, Jazan University, Jazan 45142, Saudi Arabia

[3] Department of Theoretical and Applied Chemistry, South Ural State University, Lenin prospect 76, Chelyabinsk, 454080, Russian Federation

[4] Department of Health and Biological Sciences, Abasyn University, Peshawar 25000, Pakistan

[5] School of Sciences, Yokohama City University, 22-2, Seto, Kanazawa-ku, Yokohama 236-0027, Japan

* Correspondence: Correspondence: akawsarabe@yahoo.com; Tel.: +88 0176 2717081

Citation: Ahmmed, F.; Islam, A.U.; Mukhrish, Y.E.; El Bakri, Y.; Ahmad, S.; Ozeki, Y.; Kawsar, S.M.A. Efficient Antibacterial/Antifungal Activities: Synthesis, Molecular Docking, Molecular Dynamics, Pharmacokinetic, and Binding Free Energy of Galactopyranoside Derivatives. *Molecules* **2023**, *28*, 219. https://doi.org/10.3390/molecules28010219

Academic Editor: Andreas Tzakos

Received: 3 October 2022
Revised: 13 December 2022
Accepted: 23 December 2022
Published: 26 December 2022

Copyright: © 2022 by the authors. Submitted for possible open access publication under the terms and conditions of the Creative Commons Attribution (CC BY) license (https://creativecommons.org/licenses/by/4.0/).

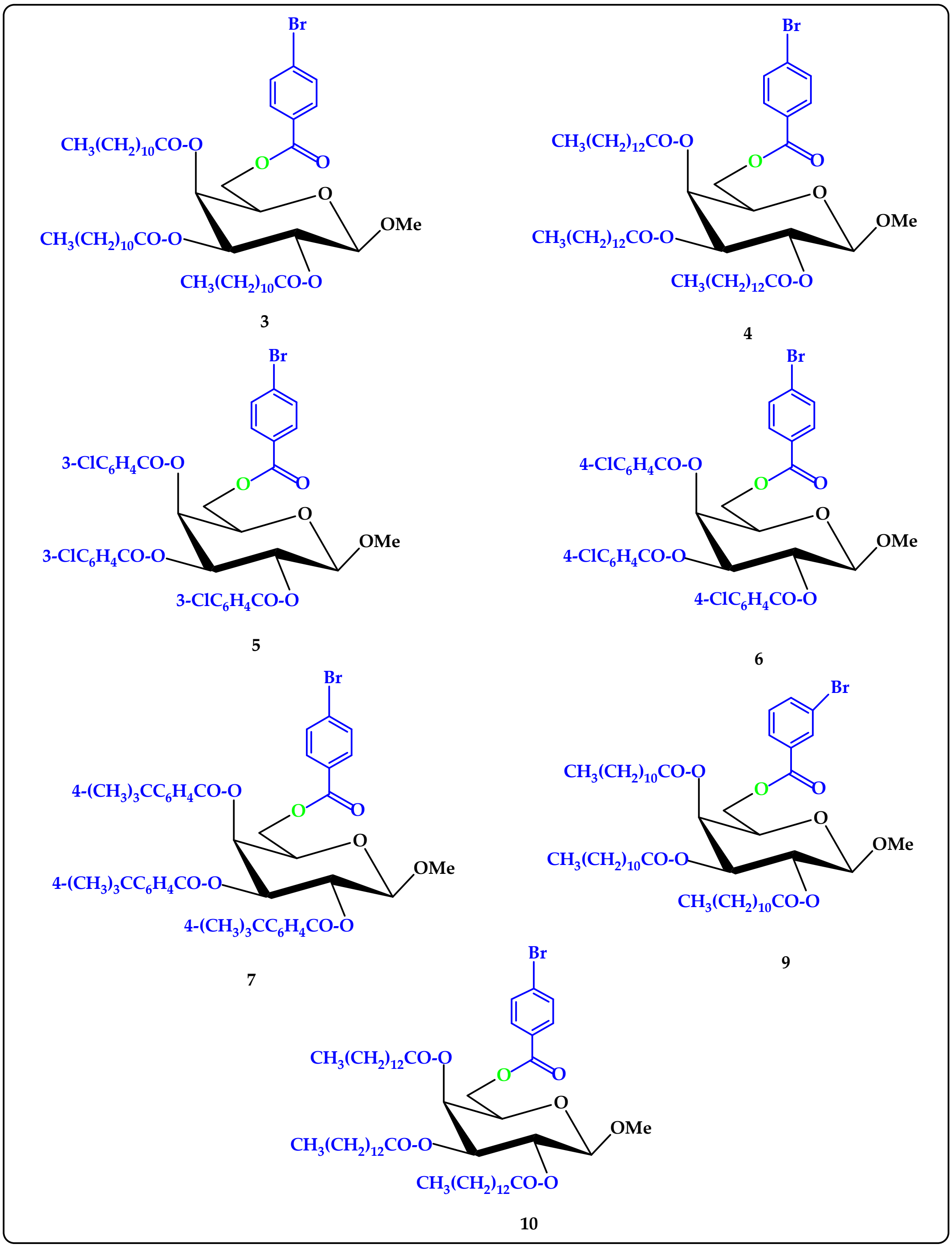

Figure S1. Chemical structures of the synthesized β-MGP derivatives (**3-10**).

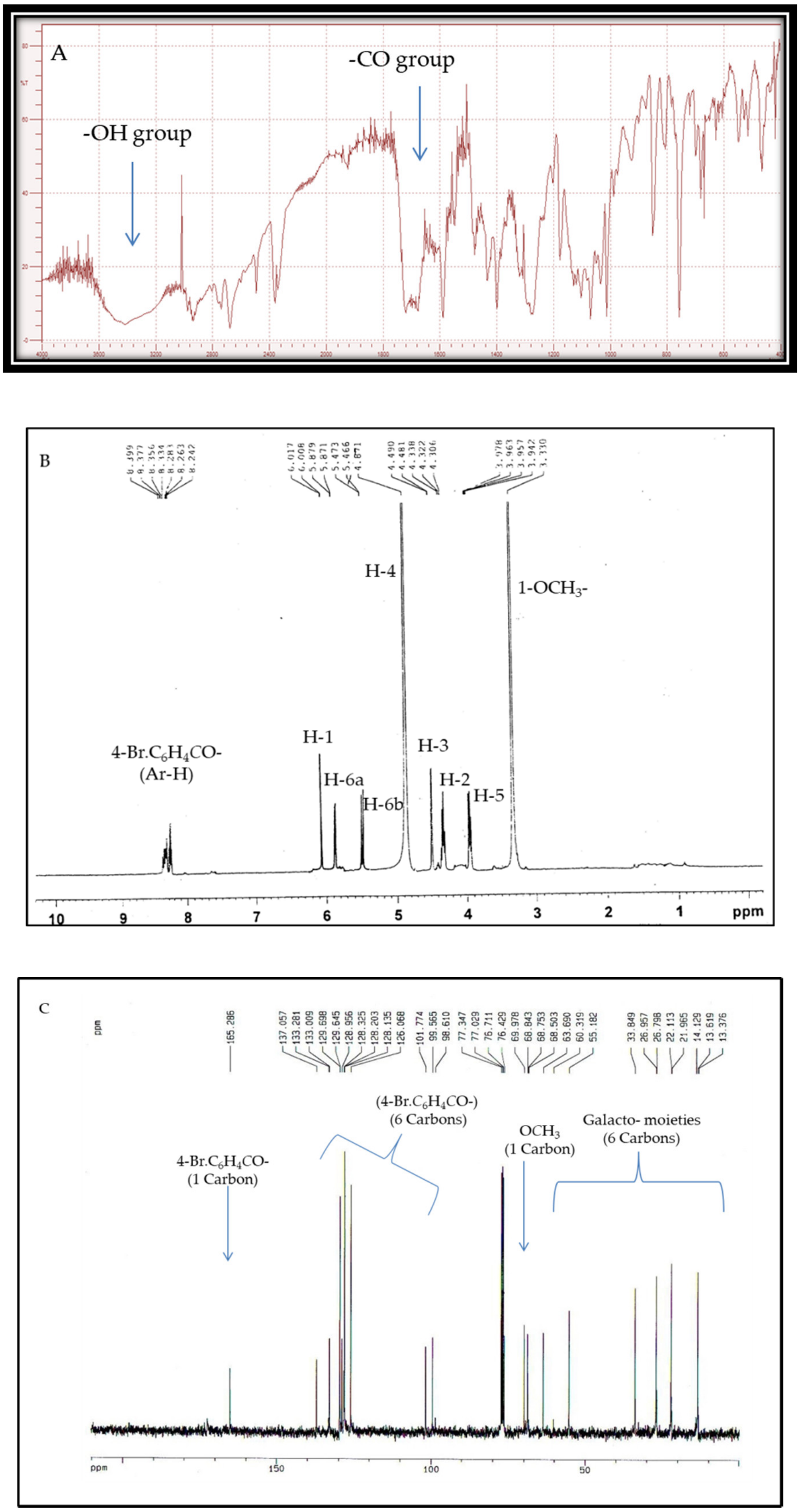

Figure S2. (A) FTIR (B) ^{1}H-NMR and (C) ^{13}C-NMR spectra of the compound **2**.

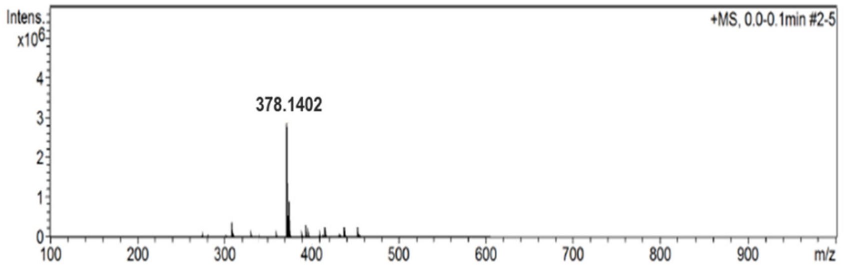

Figure S3. MS spectra of the compound **2**.

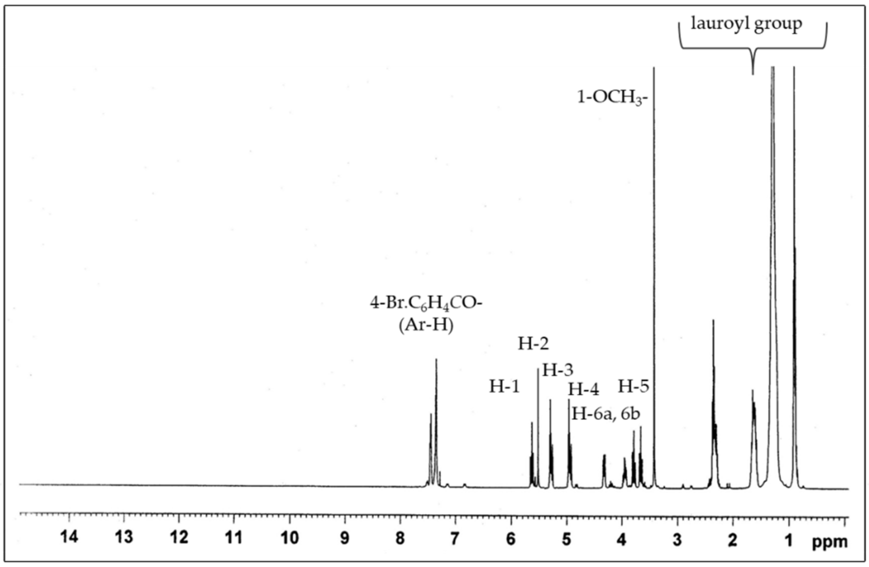

Figure S4. ^{1}H-NMR spectra of the compound **3**.

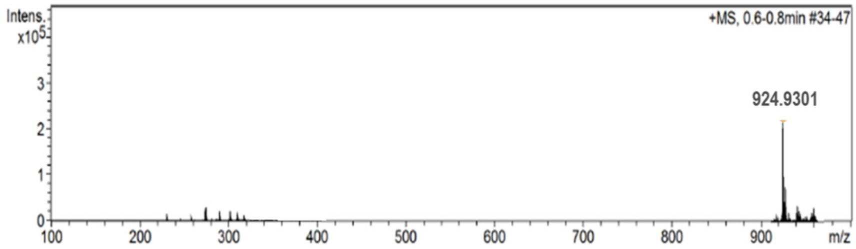

Figure S5. MS spectra of the compound **3**.

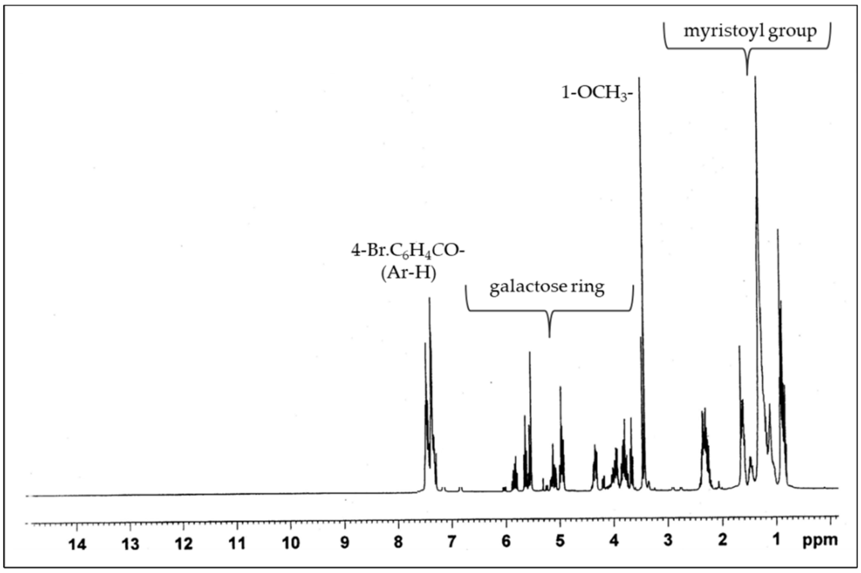

Figure S6. ^{1}H-NMR spectra of the compound **4**.

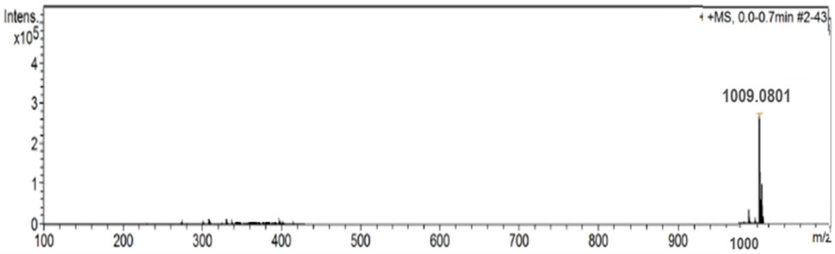

Figure S7. MS spectra of the compound **4**.

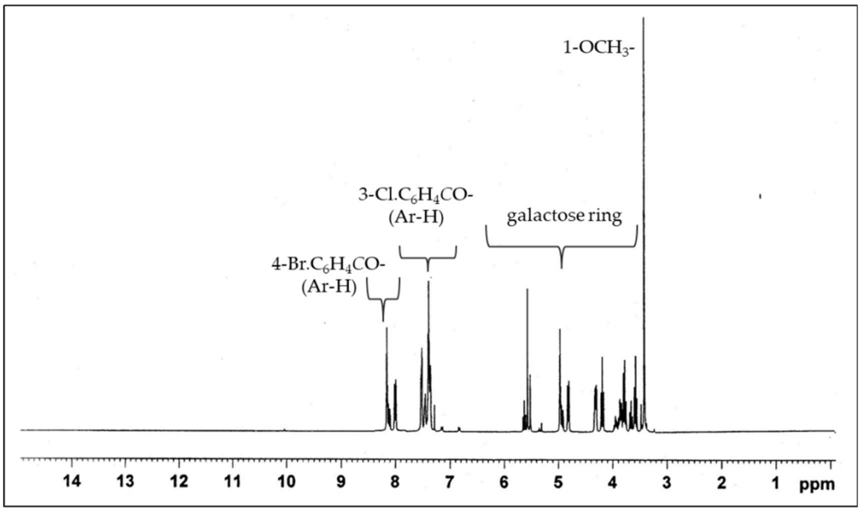

Figure S8. ^{1}H-NMR spectra of the compound **5**.

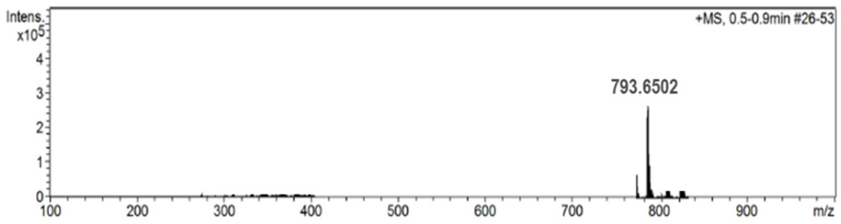

Figure S9. MS spectra of the compound **5**.

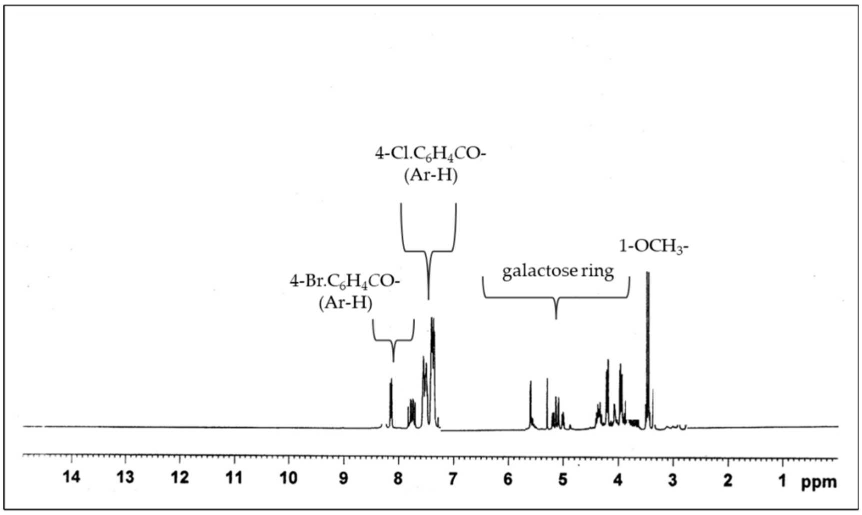

Figure S10. ^{1}H-NMR spectra of the compound **6**.

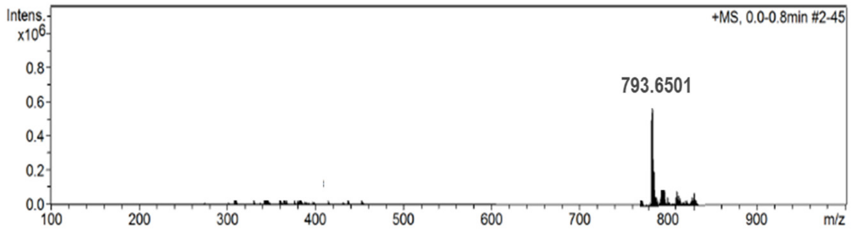

Figure S11. MS spectra of the compound **6**.

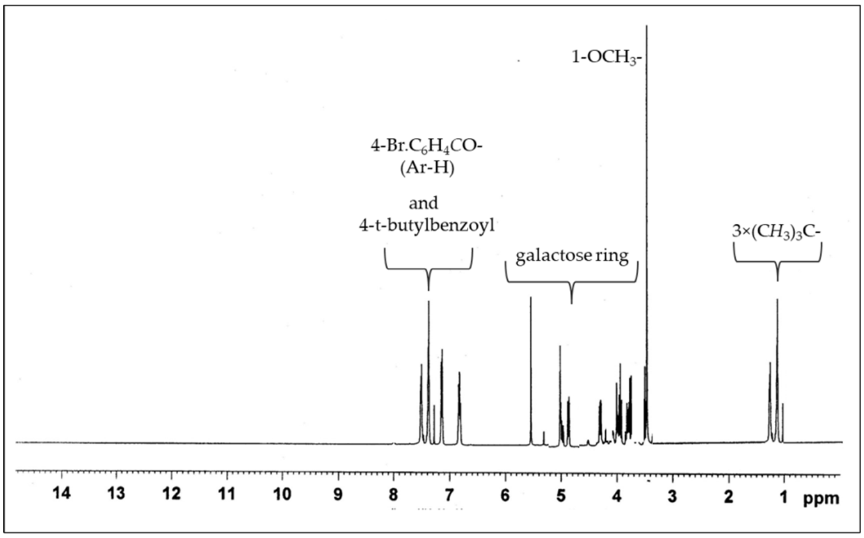

Figure S12. ^{1}H-NMR spectra of the compound **7**.

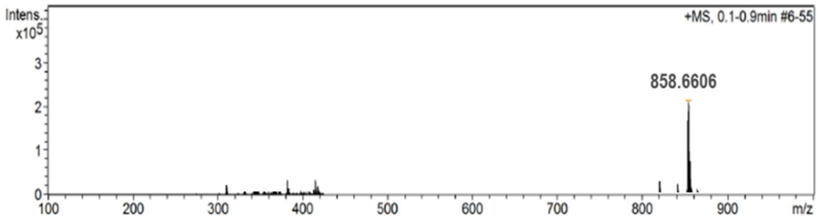

Figure S13. MS spectra of the compound **7**.

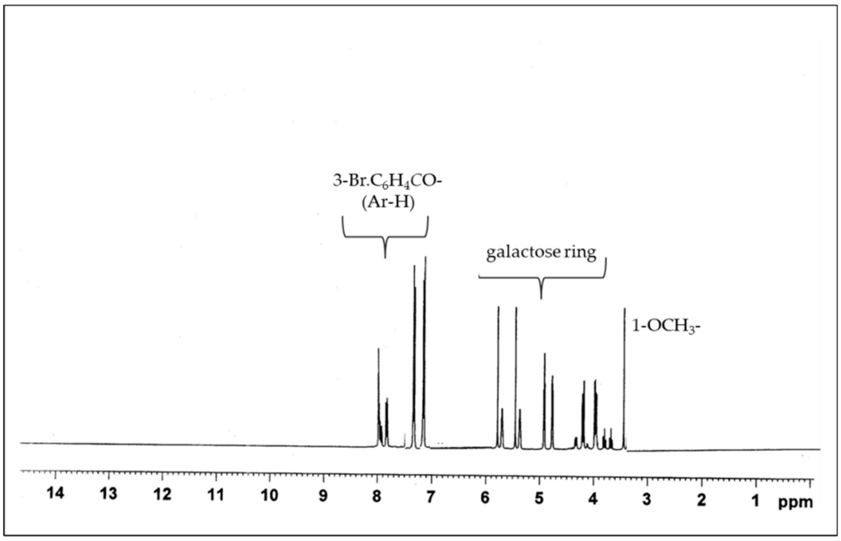

Figure S14. ^{1}H-NMR spectra of the compound **8**.

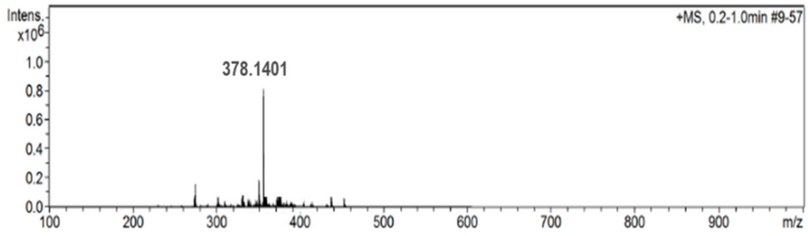

Figure S15. MS spectra of the compound **8**.

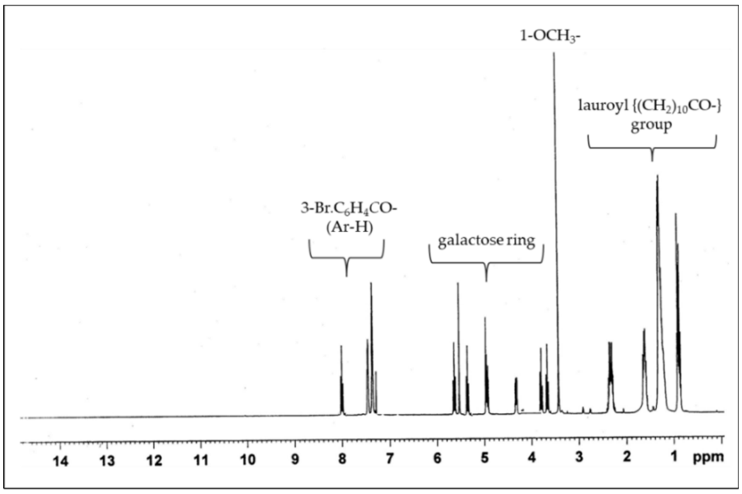

Figure S16. ^{1}H-NMR spectra of the compound **9**.

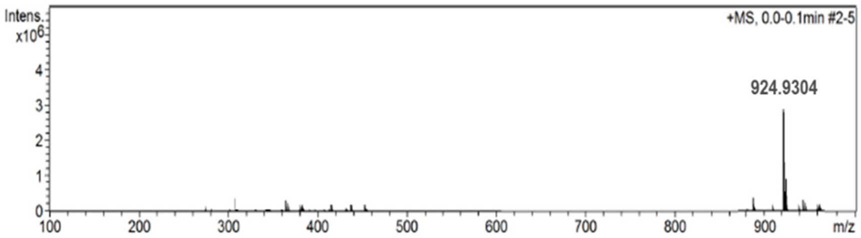

Figure S17. MS spectra of the compound **9**.

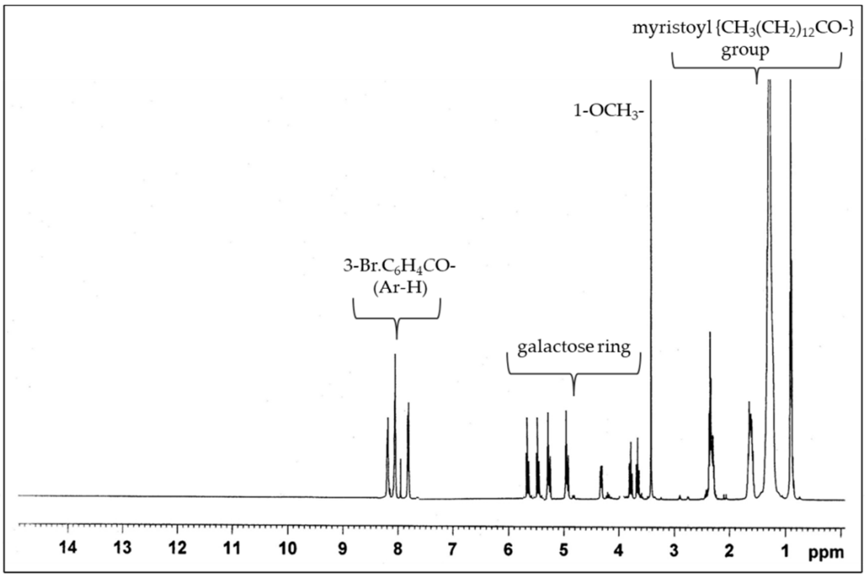

Figure S18. ^{1}H-NMR spectra of the compound **10**.

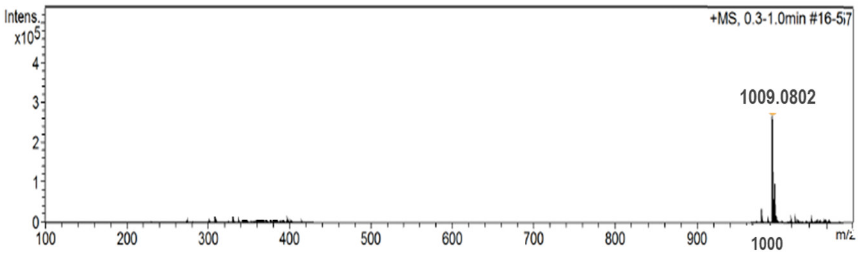

Figure S19. MS spectra of the compound **10**.

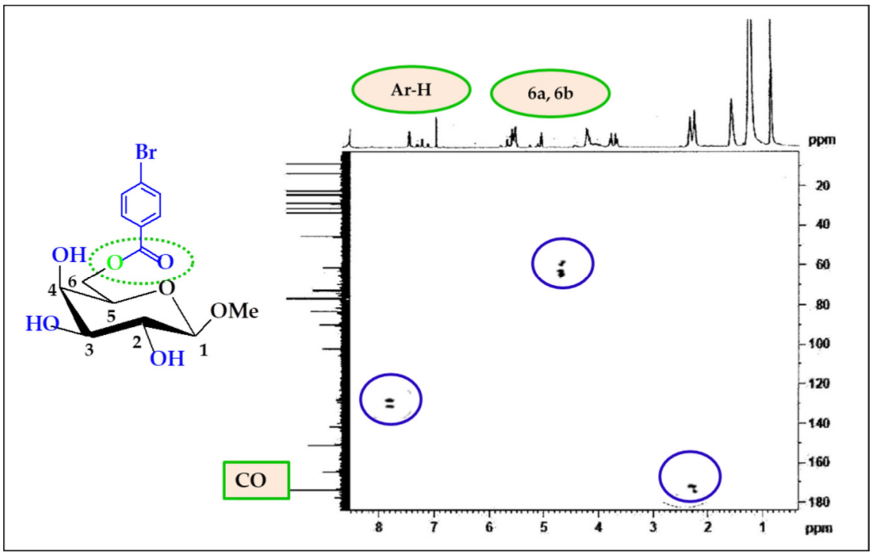

Figure S20. HMBC correlations of derivative **2**; CO with Ar-H, H-6b, and H-6b protons.

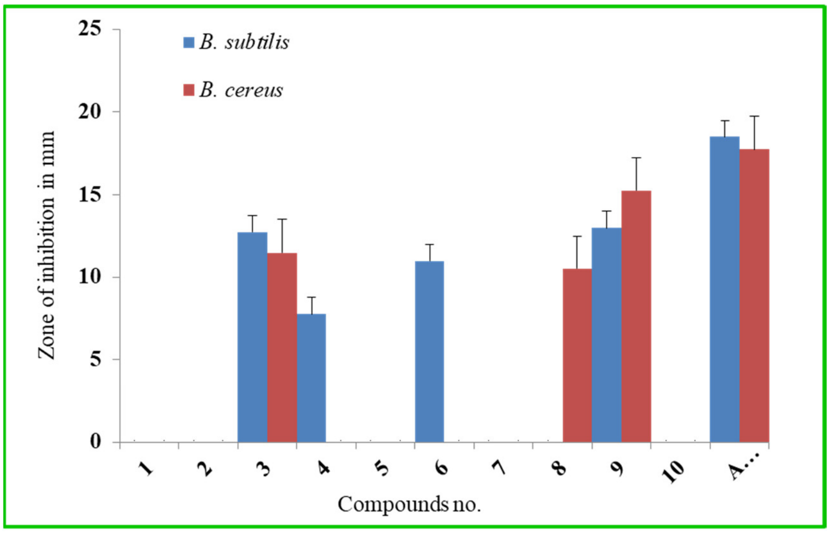

Figure S21. Zone of inhibition observed against Gram-positive bacteria by derivatives **2-10.**

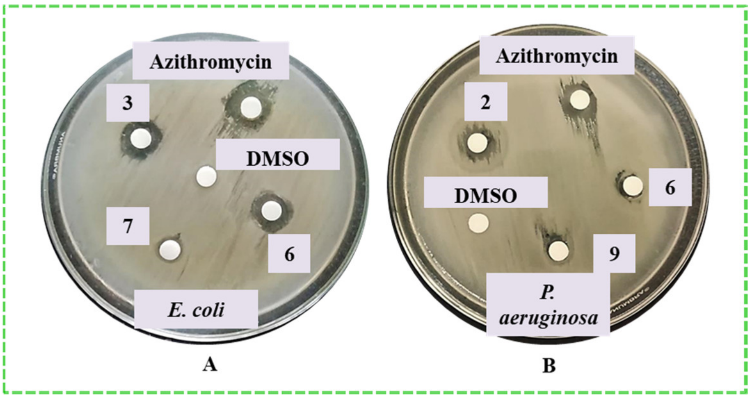

Figure S22. Experimental dishes of the synthesized derivatives **2**, **3, 6 7** and **9** against (**A**); *E. coli* and (**B**); *P. aerruginosa*, Here DMSO = Negative control and Azithromycin = Positive control.

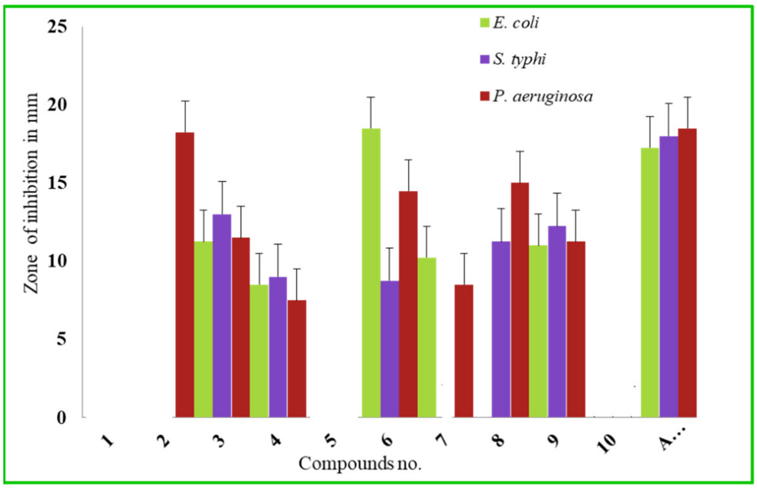

Figure S23. Zone of inhibition observed against Gram-negative bacteria by derivatives **2-10.**

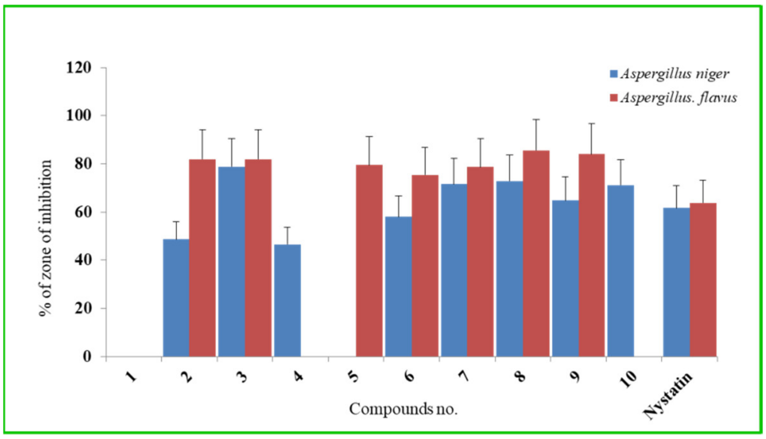

Figure S24. Antifungal activities of the synthesized derivatives **2-10**.

Table S1. ^{1}H NMR and ^{13}C NMR shift values of compound (**2**). ^{1}H and ^{13}C assignments were obtained from HSQC and HMBC experiments.

Position	δH (ppm) (J Hz)	(HSQC) δC (ppm)	HMBC
Ar-H	7.90 (m)	135.22	H: Ar
Ar-H	7.58 (m)	130.80	H: Ar
H-1	5.01 (d, J = 8.1)	101.05	H: 2, OCH_3
H-6a	4.85 (dd, J = 11.0 and 6.3)	63.31	H: 5, CO
H-6b	4.63 (dd, J = 11.0 and 6.2)	64.65	H: 5, CO
H-4	4.21 (d, J = 3.7)	76.64	H: 3, 5
H-3	4.01 (dd, J = 3.2 and 10.4)	75.08	H: 2, 4
H-2	3.92 (dd, J = 8.0 and 10.1)	68.32	H: 1, 3
H-5	3.61 (m)	58.12	H: 4, 6a, 6b
1-O*CH*$_3$	3.16 (s)	57.16	H: 1
4-Br.C_6H_4CO-		178.44	H: 6a, 6b

Table S2. The MIC and MBC values in mg/L of analogs **2** and **3** against tested organisms.

Name of bacteria	MIC (mg/L)		MBC (mg/L)	
	**Compound 3**	**Compound 9**	**Compound 3**	**Compound 9**
B. subtilis	0.125	1.00	8.00	16.00
B. cereus	2.00	0.50	16.00	8.00
E. coli	8.00	8.00	16.00	8.00
S.typhi	0.25	1.00	8.00	16.00
P. aeruginosa	2.00	2.00	8.00	16.00

Table S3. Molecular formula, molecular weight, electronic energy (*E*), enthalpy (*H*), Gibb's free energy (*G*) in Hartree and dipole moment (*p*, Debye) of β-MGP derivatives.

Entry	MF	MW	*E*	*H*	*G*	*p*
**1**	$C_7H_{14}O_6$	194.18	-722.2093	-722.2084	-722.2608	4.771
**2**	$C_{14}H_{17}O_7Br$	377.18	-3625.756	-3625.755	-3625.837	5.569
**3**	$C_{50}H_{83}O_{10}Br$	924.09	-5253.322	-5253.321	-5253.520	4.263
**4**	$C_{56}H_{95}O_{10}Br$	1008.25	-5487.767	-5487.766	-5487.984	3.321
**5**	$C_{35}H_{26}O_{10}BrCl_3$	792.84	-6025.313	-6025.312	-6025.446	7.450
**6**	$C_{35}H_{26}O_{10}BrCl_3$	792.84	-6025.325	-6025.324	-6025.445	8.574
**7**	$C_{47}H_{53}O_{10}Br$	857.82	-5121.980	-5121.979	-5121.138	7.120
**8**	$C_{14}H_{17}O_7Br$	377.14	-3625.365	-3625.364	-3625.698	5.357
**9**	$C_{50}H_{83}O_{10}Br$	923.93	-5253.192	-5253.191	-5253.456	4.478
**10**	$C_{56}H_{95}O_{10}Br$	1008.08	-5487.547	-5487.546	-5487.883	4.190

Table S4. Prediction of *in silico* of metabolism of β-MGP analogs.

Drugs	Cyp1A2	Cyp2C19	Cyp2D6	Cyp3A4
**1**	No	No	No	No
**2**	No	No	No	No
**3**	No	No	No	Yes
**4**	No	No	No	No
**5**	No	No	No	No
**6**	No	No	No	No
**7**	No	No	No	No
**8**	No	No	No	No
**9**	No	No	No	Yes
**10**	No	No	No	No

Table S5. Prediction *in silico* of the toxicity of β-MGP analogs.

Entry	Ames toxicity	T.Pyriformis Toxicity	Herg1 inhibition	LD50	Skin sensitisation
**1**	No	0.184	No	2.533	No
**2**	No	0.178	No	1.074	No
**3**	No	0.252	No	2.620	No
**4**	No	0.336	No	2.457	No
**5**	No	0.488	No	2.606	No
**6**	No	0.173	No	2.291	No
**7**	No	0.202	No	2.346	No
**8**	No	0.294	No	2.841	No
**9**	No	0.431	No	2.132	No
**10**	No	0.363	No	2.481	No

Table S6. Name of the pathogenic microorganisms.

Types of organisms	Strain	Reference
**Gram-positive bacteria**	*Bacillus subtilis*	ATCC 6633
	Bacillus cereus	BTCC 19
**Gram-negative bacteria**	*Escherichia coli*	ATCC 8739
	Salmonella typhi	AE 14612
	Pseudomonas aeruginosa	ATCC 9027
**Name of the fungi**	*Aspergillus niger*	ATCC 16404
	Aspergillus flavus	ATCC 204304

Synthesis

Methyl 6-*O*-(4-bromobenzoyl)-β-D-galactopyranoside (**2**): Yield %79.55; m.p. 67–68 °C; IR (KBr): ν/cm^{-1} 1716 (C=O), 3392~3497 (br) (-OH); ^{1}H-NMR (400 MHz, $CDCl_3$): δ_H 8.37 (2H, m, Ar-H*),* 8.26 (2H, m, Ar-H), 6.01 (1H, d, J = 8.0 Hz, H-1), 5.87 (1H, dd, J = 11.0 and 6.1 Hz, H-6a), 5.46 (1H, dd, J = 11.1 and 6.2 Hz, H-6b), 4.87 (1H, d, J = 3.6 Hz, H-4),

4.48 (1H, dd, J = 3.0 and 10.2 Hz, H-3), 4.32 (1H, dd, J = 8.1 and 10.0 Hz, H-2), 3.96 (1H, m, H-5), **3.33** (3H, s, 1-OC*H*3); MS [m/z]: 378.1402; Calcd. For $C_{14}H_{23}O_7Br$: C, 44.55%, H, 4.54%; Found: C, 44.56%, H, 4.56%.

General procedure for the preparation of lauroy derivatives ***3-7***

Methyl 6-*O*-(4-bromobenzoyl)-2,3,4-tri-*O*-lauroyl-β-D-galactopyranoside (**3**): Yield %73.71; m.p. 52-53 ^{0}C; IR (KBr): ν/cm^{-1} 1701 (C=O); ^{1}H-NMR (400 MHz, $CDCl_3$): δ_H 7.45 (2H, m, Ar-H*)*, 7.36 (2H, m, Ar-H), 5.63 (1H, d, J = 8.0 Hz, H-1), 5.61 (1H, dd, J = 8.0 and 10.2 Hz, H-2), 5.58 (1H, dd, J = 3.1 and 10.5 Hz, H-3), 4.97 (1H, d, J = 3.5 Hz, H-4), 4.33 (1H, dd, J = 11.1 and 6.2 Hz, H-6a), 3.79 (1H, dd, J = 11.1 and 6.3 Hz, H-6b), 3.66 (1H, m, H-5), 3.42 (3H, s, 1-OCH_3), 2.33 {6H, m, 3×$CH_3(CH_2)_9CH_2$CO-}, 1.63 {6H, m, 3×$CH_3(CH_2)_8CH_2CH_2$CO-}, 1.28 {48H, m, 3×$CH_3(CH_2)_8CH_2CH_2$CO-}, 0.88 {9H, m, 3×$CH_3(CH_2)_{10}$CO-}; MS [m/z]: 924.9301; Calcd. For $C_{50}H_{83}O_{10}Br$: C, 64.94%, H, 9.05%; Found: C, 64.96%, H, 9.06%.

Methyl 6-*O*-(4-bromobenzoyl)-2,3,4-tri-*O*-myristoyl-β-D-galactopyranoside (**4**): Yield %75.89; Mp: 54-56°C (EtOAc-*n*-C_6H_{14}, R_f = 0.53); IR (KBr): ν/cm^{-1} 1706 (C=O); ^{1}H-NMR (400 MHz, $CDCl_3$): δ_H 7.49 (2H, m, Ar-H*)*, 7.40 (2H, m, Ar-H), 5.81 (1H, d, J = 8.1 Hz, H-1), 5.66 (1H, dd, J = 8.1 and 10.2 Hz, H-2), 5.63 (1H, dd, J = 3.1 and 10.2 Hz, H-3), 5.54 (1H, d, J = 3.2 Hz, H-4), 4.32 (1H, dd, J = 11.1 and 6.8 Hz, H-6a), 3.96 (1H, dd, J = 11.0 and 6.3 Hz, H-6b), 3.82 (1H, m, H-5), 3.42 (3H, s, 1-OCH_3), 2.31 {6H, m, 3×$CH_3(CH_2)_{11}CH_2$CO-}, 1.60 {6H, m, 3×$CH_3(CH_2)_{10}CH_2CH_2$CO-}, 1.25 {60H, m, 3×$CH_3(CH_2)_{10}CH_2CH_2$CO-}, 0.87 {9H, m, 3×$CH_3(CH_2)_{12}$CO-}; MS [m/z]: 1009.0801; Calcd. For $C_{56}H_{95}O_{10}Br$: C, 66.66%, H, 9.50%; Found: C, 66.68%, H, 9.52%.

Methyl 6-*O*-(4-bromobenzoyl)-2,3,4-tri-*O*-(3-chlorobenzoyl)-β-D-galactopyranoside (**5**): Yield %74.02; m.p. 134–136 °C (EtOAc-*n*-C_6H_{14}, R_f = 0.53); IR (KBr): ν/cm^{-1} 1692 (-CO); ^{1}H-NMR (400 MHz, $CDCl_3$): δ_H 8.09 (3H, m, Ar-H), 8.01 (3H, m, Ar-H), 7.48 (2H, m, Ar-H*)*, 7.44 (2H, m, Ar-H), 7.40 (3H, m, Ar-H), 7.35 (3H, m, Ar -H), 5.64 (1H, m, br, H-1), 5.52 (1H, dd, J = 8.1 and 10.3 Hz, H-2), 4.94 (1H, dd, J = 3.0 and 10.2 Hz, H-3), 4.80 (1H, d, J = 3.5 Hz, H-4), 4.21 (1H, dd, J = 11.1 and 6.3 Hz, H-6a), 3.84 (1H, dd, J = 11.0 and 6.1 Hz, H-6b), 3.58 (1H, m, H-5), 3.42 (3H, s, 1-OCH_3); MS [m/z]: 793.6502; Calcd. For $C_{35}H_{26}O_{10}$Br.3Cl: C, 52.99%, H, 3.31%; Found: C, 53.01%, H, 3.3%.

Methyl 6-*O*-(4-bromobenzoyl)-2,3,4-tri-*O*-(4-chlorobenzoyl)-β-D-galactopyranoside (**6**): Yield %57.85; m.p. 160-161 ^{0}C (EtOAc-*n*-C_6H_{14}, R_f = 0.56); IR (KBr): ν/cm^{-1} 1711 (-CO); ^{1}H-NMR (400 MHz, $CDCl_3$): δ_H 8.10 (6H, m, Ar-H), 7.88 (2H, m, Ar-H), 7.52 (6H, m, Ar-H), 7.42 (2H, m, Ar-H), 5.65 (1H, d, J = 3.2 Hz, H-1), 5.23 (1H, dd, J = 3.4 and 10.0 Hz, H-2), 5.10 (1H, m, H-3), 4.97 (1H, t, J = 9.1 Hz, H-4), 4.14 (1H, m, H-6a), 4.01 (1H, t, J = 10.2 Hz, H-6b), 3.98 (1H, m, H-5), 3.41 (3H, s, 1-OCH_3); MS [m/z]: 793.6501; Calcd. For $C_{35}H_{26}O_{10}$Br.3Cl: C, 52.99%, H, 3.31%; Found: C, 52.97%, H, 3.30%.

Methyl 6-*O*-(4-bromobenzoyl)-2,3,4-tri-*O*-(4-t-butylbenzoyl)-β-D-galactopyranoside (**7**): Yield %45.51; m.p. 106-107 °C (EtOAc-*n*-C_6H_{14}, R_f = 0.54); IR (KBr): ν/cm^{-1} 1716 (-CO); ^{1}H-NMR (400 MHz, $CDCl_3$) (ppm): δ_H 7.51 (2H, m, Ar-H), 7.38 (6H, m, 3×Ar-H), 7.15 (2H, m, Ar-H), 6.91 (6H, m, 3×Ar-H), 5.52 (1H, d, J = 8.1 Hz, H-1), 5.02 (1H, dd, J = 8.1 and 10.2 Hz, H-2), 4.89 (1H, dd, J = 3.2 and 10.2 Hz, H-3), 4.58 (1H, d, J = 3.3 Hz, H-4), 4.01 (1H, dd, J = 11.0 and 6.3 Hz, H-6a), 3.86 (1H, dd, J = 11.0 and 6.3 Hz, H-6b), 3.78 (1H, m, H-5), 3.42 (3H, s, 1-OCH_3), 1.07, 1.10, 1.18 {27H, 3×s, 3×(CH_3)$_3$C-}; MS [m/z]: 858.6606; Calcd. For $C_{47}H_{53}O_{10}Br$: C, 65.76%, H, 6.23%; Found: C, 65.77%, H, 6.24%.

Methyl 6-*O*-(3-bromobenzoyl)-β-D-galactopyranoside (**8**): Yield %48.25; m.p. 108-109 °C (EtOAc-*n*-C_6H_{14}, R_f = 0.50); IR (KBr): ν/cm^{-1} 1720 (C=O), 3401~3496 (br) (-OH); ^{1}H-NMR (400 MHz, $CDCl_3$): δ_H 8.01 (1H, d, J = 7.1 Hz, Ar-H), 7.95 (1H, s, Ar-H), 7.22 (1H, d, J = 7.2 Hz, Ar-H), 7.13 (1H, t, J = 7.4 Hz, Ar -H), 5.86 (1H, d, J = 8.0 Hz, H-1), 5.55 (1H, dd, J = 11.1 and 6.5 Hz, H-6a), 4.98 (1H, dd, J = 11.1 and 6.7 Hz, H-6b), 4.88 (1H, d, J = 3.5 Hz, H-4), 4.21 (1H, dd, J = 3.0 and 10.5 Hz, H-3), 3.99 (1H, dd, J = 8.0 and 10.5 Hz, H-2), 3.86 (1H, m, H-5), 3.41 (3H, s, 1-OCH_3); MS [m/z]: 378.1401; Calcd. For $C_{14}H_{17}O_7Br$: C, 44.55%, H, 4.54%; Found: C, 44.56%, H, 4.56%.

Methyl 6-*O*-(3-bromobenzoyl)-2,3,4-tri-*O*-lauroyl-β-D-galactopyranoside (**9**): Yield %68.09; m.p. 114-115 °C (EtOAc-*n*-C_6H_{14}, R_f = 0.53); IR (KBr): ν/cm^{-1} 1719 (C=O); ^{1}H-NMR (400 MHz, $CDCl_3$) (ppm): δ_H 8.01 (1H, d, J = 7.3 Hz, Ar-H), 7.47 (1H, s, Ar-H), 7.36 (1H, d, J = 7.3 Hz, Ar-H), 7.16 (1H, t, J = 7.3 Hz, Ar -H), 5.63 (1H, d, J = 8.3 Hz, H-1), 5.52 (1H, dd, J = 8.0 and 10.2 Hz, H-2), 5.49 (1H, dd, J = 3.1 and 10.1 Hz, H-3), 4.97 (1H, d, J = 3.1 Hz, H-4), 4.33 (1H, dd, J = 11.0 and 6.0 Hz, H-6a), 3.78 (1H, dd, J = 11.1 and 6.4 Hz, H-6b), 3.66 (1H, m, H-5), 3.42 (3H, s, 1-OCH_3), 2.32 {6H, m, 3×$CH_3(CH_2)_9$CH_2CO-}, 1.62 {6H, m, 3×$CH_3(CH_2)_8$C$H_2$$CH_2$CO-}, 1.25 {48H, m, 3×$CH_3$(C$H_2$)$_8$$CH_2CH_2$CO-}, 0.89 {9H, m, 3×C$H_3$$(CH_2)_{10}$CO-}; MS [m/z]: 924.9304; Calcd. For $C_{50}H_{83}O_{10}Br$: C, 64.94%, H, 9.06%; Found: C, 64.95%, H, 9.08%.

Methyl 6-*O*-(3-bromobenzoyl)-2,3,4-tri-*O*-myristoyl-β-D-galactopyranoside (**10**): Yield %73.05; m.p. 118-119 °C (EtOAc-*n*-C_6H_{14}, R_f = 0.55); IR (KBr): ν/cm^{-1} 1718 cm^{-1} (C=O); ^{1}H-NMR (400 MHz, $CDCl_3$) (ppm): δ_H 8.11 (1H, d, J = 7.3 Hz, Ar-H), 8.04 (1H, s, Ar-H), 7.96 (1H, d, J = 7.3 Hz, Ar-H), 7.86 (1H, t, J = 7.3 Hz, Ar -H), 5.69 (1H, d, J = 8.1 Hz, H-1), 5.53 (1H, dd, J = 8.1 and 10.2 Hz, H-2), 5.28 (1H, dd, J = 3.1 and 10.2 Hz, H-3), 4.97 (1H, d, J = 3.2 Hz, H-4), 4.29 (1H, dd, J = 11.1 and 6.8 Hz, H-6a), 3.83 (1H, dd, J = 11.0 and 6.3 Hz, H-6b), 3.62 (1H, m, H-5), 3.42 (3H, s, 1-OCH_3), 2.24 {6H, m, 3×$CH_3(CH_2)_{11}$CH_2CO-}, 1.63 {6H, m, 3×$CH_3(CH_2)_{10}$C$H_2$$CH_2$CO-}, 1.26 {60H, m, 3×$CH_3$(C$H_2$)$_{10}$$CH_2CH_2$CO-}, 0.88 {9H, m, 3×C$H_3$$(CH_2)_{12}$CO-}; MS [m/z]: 1009.0802; Calcd. For $C_{56}H_{95}O_{10}Br$: C, 66.66%, H, 9.50%; Found: C, 66.68%, H, 9.52%.
